# Thyroid orbitopathy

**DOI:** 10.1590/0100-3984.2024.57.e13-en

**Published:** 2024-12-27

**Authors:** Murilo Bicudo Cintra

**Affiliations:** 1 Radiologist at the Instituto do Câncer do Estado de São Paulo (ICESP) and for the Grupo Dasa, São Paulo, SP, Brazil. Email: bicudocintra@gmail.com

Graves’ disease is an autoimmune condition and the most common cause of hyperthyroidism in countries with iodine sources. In this disease, B lymphocytes produce high levels of thyroid-stimulating hormone receptor antibodies, which bind to those receptors in the thyroid gland, promoting glandular enlargement and hypersecretion of thyroid hormones. In the general population, Graves’ disease has a prevalence of 1.0-1.5%, with an annual incidence of approximately 20-30 new cases per 100,000 population. Graves’ disease mainly affects individuals between 30 and 60 years of age, is 5-10 times more common in women, and has a higher incidence among individuals of African descent. Genetic predisposition carries a risk of up to 79% of developing the disease, whereas environmental factors account for a risk of approximately 21%^(1,2)^.

Graves’ orbitopathy occurs in approximately 20-25% of individuals with Graves’ disease and is in the moderate-to-severe form in 4.9% of such individuals. The pathophysiology is related to a common antigen of the disease in the thyroid and orbital structures. Ophthalmopathy is the most common extra-thyroidal manifestation of Graves’ disease and presents an initial inflammatory phase, followed by a plateau or stabilization phase, and eventually evolves to an inactive phase. Mild cases tend to resolve spontaneously, although complete resolution almost never occurs in moderate and severe cases.

Early diagnosis, disease control, and removal of external risk factors can effectively limit the progression of Graves’ orbitopathy, having a significant impact on the quality of life of the affected individuals. Symptoms range from eye irritation to vision loss. The initial diagnosis is based on clinical and laboratory data. Imaging tests are important to assess orbital changes and are useful for assessing disease progression and for surgical planning, as well as for excluding differential diagnoses. They can also provide valuable information regarding factors such as increased volume of adipose tissue, thickening of extraocular muscles, and compression of the optic nerve^(3)^.

The article “Morphometric analysis of the extraocular musculature and proptosis by computed tomography in Graves’ orbitopathy”, recently published in **Radiologia Brasileira**^(4)^, demonstrates that in computed tomography examinations of the orbits it is possible to establish a correlation between the severity of the orbitopathy and the increase in the thickness of the extraocular muscles and the degree of proptosis, by evaluating those structures, indicating that muscle thickening provides a possible future direction for research, because it has the potential to be a biomarker of the risk of vision loss. The authors drew their conclusions through simple measurements of the thickness of the extraocular muscles and the degree of proptosis. As a limitation of the article, we emphasize the need for validation in the literature, at other centers and, if possible, in prospective studies involving larger samples.

The main imaging methods evaluated in the literature for the diagnosis, monitoring, and prognosis of thyroid orbitopathy include computed tomography, magnetic resonance imaging and positron emission tomography/computed tomography, as well as Doppler ultrasound. As a common factor, most studies include morphological evaluation through measurements of the extraocular muscles, together with evaluation of the measurements of the eyeball, lacrimal glands, optic nerves, and orbital fat. These measurements, mainly those obtained by magnetic resonance imaging and computed tomography, are similar across studies. However, each method attempts to highlight evolutionary, inflammatory, and prognostic information addressing specific characteristics including diffusion of water molecules, signal intensity ratio between the brain and extraocular muscles, in addition to evaluating fibrosis and dynamic contrast changes in magnetic resonance imaging studies. These data allow the evaluation of microstructural changes (diffusion) and disease activity (signal ratio), thus contributing to the choice of the best treatment and monitoring in routine examinations. Through the use of diffusion tensor imaging sequences, magnetic resonance imaging has greater sensitivity for detecting changes in the optic nerves^(3)^.

It is noteworthy that there have been studies using color Doppler ultrasound to evaluate the superior ophthalmic vein, the ophthalmic artery, and the central retinal artery, as well as the regional microvasculature. Changes in orbital vascular congestion during the progression of the disease, as evidenced by reduced flow in the superior ophthalmic vein, together with high flow and peak velocity of the ophthalmic artery and central retinal artery, can reveal disease activity^(5)^.

## References

[1] Antonelli A, Ferrari SM, Ragusa F (2020). Graves’ disease: epidemiology, genetic and environmental risk
factors and viruses. Best Pract Res Clin Endocrinol Metab.

[2] Lanzolla G, Marinò M, Menconi F (2024). Graves disease: latest understanding of pathogenesis and
treatment options. Nat Rev Endocrinol.

[3] Luccas R, Riguetto CM, Alves M (2024). Computed tomography and magnetic resonance imaging approaches to
Graves’ ophthalmopathy: a narrative review. Front Endocrinol (Lausanne).

[4] Watanabe EM, Cavazzana RY, Ribeiro DAM (2024). Análise morfométrica da musculatura ocular
extrínseca e da proptose por tomografia computadorizada na
orbitopatia de Graves. Radiol Bras.

[5] Goel R, Shah S, Sundar G (2023). Orbital and ocular perfusion in thyroid eye
disease. Surv Ophthalmol.

